# Perception of partial slips under tangential loading of the fingertip

**DOI:** 10.1038/s41598-018-25226-w

**Published:** 2018-05-04

**Authors:** Allan Barrea, Benoit P. Delhaye, Philippe Lefèvre, Jean-Louis Thonnard

**Affiliations:** 1 Institute of Neuroscience, Université catholique de Louvain, Brussels, B-1200 Belgium; 2 ICTEAM Institute, Université catholique de Louvain, Louvain-la-Neuve, B-1348 Belgium; 3 Physical and Rehabilitation Medicine Department, Cliniques Universitaires Saint-Luc, Brussels, B-1200 Belgium; 4 Department of Organismal Biology and Anatomy, University of Chicago, Chicago, Illinois United States

## Abstract

During tactile exploration, partial slips occur systematically at the periphery of fingertip-object contact prior to full slip. Although the mechanics of partial slips are well characterized, the perception of such events is unclear. Here, we performed psychophysical experiments to assess partial slip detection ability on smooth transparent surfaces. In these experiments, the index fingertip of human subjects was stroked passively by a smooth, transparent glass plate while we imaged the contact slipping against the glass. We found that subjects were able to detect fingertip slip before full slip occurred when, on average, only 48% of the contact area was slipping. Additionally, we showed that partial slips and plate displacement permitted slip detection, but that the subjects could not rely on tangential force to detect slipping of the plate. Finally, we observed that, keeping the normal contact force constant, slip detection was impeded when the plate was covered with a hydrophobic coating dramatically lowering the contact friction and therefore the amount of fingertip deformation. Together, these results demonstrate that partial slips play an important role in fingertip slip detection and support the hypothesis that the central nervous system relies on them to adjust grip force during object manipulation.

## Introduction

Our tactile sense provides a wealth of information about our environment. Stroking our fingertips over a surface allows us to perceive many physical properties, including texture^1^, relative speed between the fingertip and the surface^2,3^, friction^4^, and submicron-scale features^5,6^. Thus, mechanical interactions between finger pads and the surfaces they contact play a critical role in tactile perception.

When a fingertip is loaded tangentially against a surface, slippage occurs progressively with a very reproducible two-phase pattern. In the first phase, called incipient slip, slippage occurs at the periphery of contact. The no-slip area in the centre of the contact area decreases gradually with increasing loading until gross slippage occurs. In the second phase, called full slip, the entire contact area slips. The biomechanics of the partial slip phenomenon has been described thoroughly *in vivo* in passive conditions^7–11^ and modelled mathematically within the framework of contact mechanics for an ideal elastic sphere^12^. Although a fingertip is not an ideal elastic sphere, the model predictions were qualitatively confirmed *in vivo*^10,11^.

Stick ratio (SR) was proposed to quantify partial slip at the fingertip contact^8,9^. SR is defined as the ratio of the no-slip area to the entire contact area. SR falls progressively from 1 (no slip) to 0 (full slip) as partial slip develops within the contact, starting from the periphery of the contact and propagating towards its centre until there is a full slip.

Srinivasan *et al*. demonstrated the capacity of tactile afferents to encode stimulus features related to fingertip slip^13^. They performed psychophysical experiments wherein human subjects detected the relative motion (i.e. slip) of plates stroked under their fingertip. In addition, they recorded the responses evoked in tactile afferent fibres of macaque monkeys exposed to the same paradigm. The human subjects detected loading direction but not slippage of a smooth glass plate. Importantly, the plate was pressed against the fingertip with a very low normal force (NF), inducing very little fingertip deformation. However, when micrometre-sized protrusions were added to the glass, subjects were able to detect the plate slipping. The firing rate of rapidly adapting afferent fibres innervating the hand of monkeys increased when they were exposed to the non-smooth plates. The authors concluded that surface protrusion-induced vibrations generated in the skin by the non-smooth plates caused the increased firing rate, which in turn helped the central nervous system detect slippage. Similarly, Johansson and Westling had demonstrated previously that rapidly adapting afferent fibres are responsive to fingertip slip during object grasping^14^.

**Figure 1:**
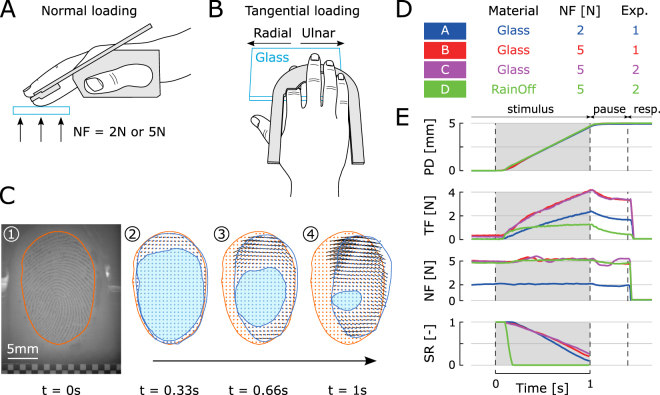
Apparatus, experimental conditions and typical traces. **(A)** The right index fingertip of the subject (side view) is kept in place with a rigid support (in grey). A glass plate (blue) is pressed vertically against it with a controlled NF of 2N or 5N. **(B)** The glass plate is then moved horizontally with a speed of 5 mm/s in the radial or ulnar direction before retracting from the fingertip (top view). **(C**.**1)** Typical fingerprint image acquired with the imaging system composed of a coaxial light source and camera. The orange contour delimits the contact between the fingertip and the plate. **(C**.**2–C**.**4)** Illustration of finger pad deformation under ulnar loading at constant speed (5 mm/s) over a distance of 5 mm with NF = 2N (corresponding to blue curves in panel E). The orange contour is the initial contact area reproduced from subpanel C.1. The blue contour is the contact area imaged at different times. The no-slip area (light blue) decreases with increasing tangential loading, while the periphery of the contact area experiences slippage. Orange dots sampled regularly in the initial contact area are linked with black lines to corresponding blue dots in the contact area under loading. **(D)** Summary of experimental conditions. Two stimuli were used: a bare glass plate (“Glass”) and a glass plate covered with a hydrophobic solution strongly reducing the surface-skin friction (“RainOff”). **(E)** Typical traces from subject S01 with a total displacement of 5 mm in the ulnar direction: plate displacement (PD), tangential force (TF), normal force (NF) and stick ratio (SR). The grey window represents the time period during which the robot was receiving a tangential displacement command and fingerprint images were recorded. Subjects’ responses were collected at the end of each trial, i.e. after the plate was retracted from the finger pad. Colours correspond to experimental conditions described in panel D.

To our knowledge, no prior study has examined the perception of fingertip slip while simultaneously imaging incipient slip and measuring fingertip contact dynamics. Here, we conducted two psychophysical experiments in which the right index fingertip of human subjects was stroked passively with a speed of 5 mm/s by a smooth, transparent glass plate in successive trials (Fig. 1A,B). After each trial, we asked the subjects whether they felt the plate slip under their fingertip or not. At the same time, we quantified the amount of partial slip taking place between the fingertip and the plate by imaging the finger pad through the transparent plate (Fig. 1C). Plate displacement was varied randomly across trials and ranged between a small displacement corresponding to almost no slip to a large displacement bringing the fingertip contact close to full slip. In the first experiment, we contrasted two levels of NF (2N vs. 5N) and in the second one, we contrasted two levels of friction (high vs. low). The experimental conditions are summarized in Fig. 1D.

We interpreted subjects’ responses against three different predictors: plate displacement (PD), stick ratio (SR), and tangential force (TF) applied to the plate by the fingertip. Predictor values were sampled at the end of each trial. Psychometric curves were fitted on subjects’ answers, allowing us to extract the slip detection threshold for each one of the three predictors. Each threshold was defined as the value of the corresponding predictor for which the subject felt the plate slip 50% of the time. To determine which predictor underlay slip detection, thresholds were compared across experimental conditions. If the threshold was consistent across conditions, then we inferred that the predictor could be a cue for slip detection. In contrast, if the threshold differed significantly across conditions, then we inferred that the predictor was ambiguous and not a useful cue for slip detection.

## Results

### Experiment 1: NF contrast

**Figure 2:**
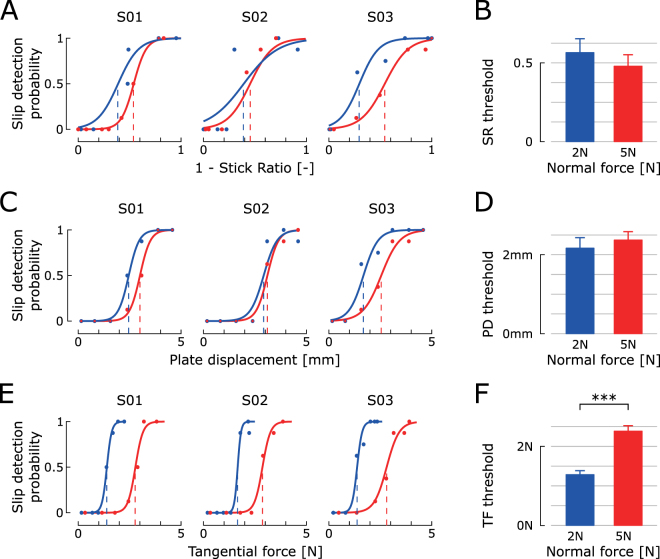
Experiment 1: Slip detection thresholds on bare glass with two NF levels. Subjects detected fingertip slip before full slip and could not rely on TF to detect slip. Slip detection responses from three typical subjects are plotted against 1 – SR, PD, and TF in panels A, C and E, respectively. Psychometric curves were fitted on subjects’ answers from the two experimental conditions (NF = 2 N, blue, vs. 5N, red), which allowed computation of slip detection thresholds, i.e. predictor values corresponding to a 50% probability of slip detection. Thresholds from all subjects were compared between the two conditions in panels B,D, and F for SR, PD, and TF, respectively. Bar charts summarize the thresholds in each condition as means ± standard errors across subjects. Remarkably, subjects detected slip before full slip in both conditions. Slip detection thresholds for TF, but not for PD and SR, varied across conditions, indicating that TF was unreliable for slip detection. For readability, subjects’ answers are plotted against 1 – SR in panel (A), such that increasing values on the x-axis correspond to greater partial slip. In panels A, C and E, the coordinates of blue and red dots were computed as the average of all trials with the same PD within a given condition. They are displayed for illustrative purposes only; psychometric curves were fitted on data from individual trials.

The first experiment contrasted two NF levels: 2N vs. 5N. Figure 2A presents the responses of three typical subjects. Data are presented from stick to slip and are therefore plotted against 1 – SR. This quantity monotonically increases from 0 to 1 between full stick and full slip and thus represents the actual slippage level between the fingertip and the plate. The subjects perceived fingertip slip before the actual full slip, i.e. before SR = 0, consistently under both the 2N and 5N NF conditions. The average slip detection threshold across all participants in the two conditions corresponded to a SR of 0.52 (95% confidence interval (CI) = [0.4; 0.64]). The detection thresholds from Fig. 2A are summarized in Fig. 2B for all subjects; no significant difference was found between the two NF conditions (paired t-test, t(9) = 2.12, p > 0.05). Similarly, PD threshold did not differ between the two NF conditions (paired t-test, t(9) = 1.52, p > 0.05; Fig. 2C,D). The average slip detection threshold across all participants in the two NF conditions corresponded to a PD of 2.26 mm (95% CI = [1.92; 2.62]). For TF (Fig. 2E,F), detection thresholds differed significantly across the two NF conditions (paired t-test, t(9) = 15.76, p < 0.001), indicating that subjects could not rely on TF to detect fingertip slip. In contrast, the threshold invariance for SR and PD suggests that subjects could rely on at least one of these predictors or a combination of both to perceive fingertip slip.

Trials were performed randomly in the ulnar or radial direction (see Methods), with an equal total number of trials in each direction, in order to prevent stimulus habituation. There was no effect of stimulation direction on subject responses (Kolmogorov-Smirnov test on individual subjects’ answers, p > 0.05).

### Experiment 2: Friction contrast

**Figure 3:**
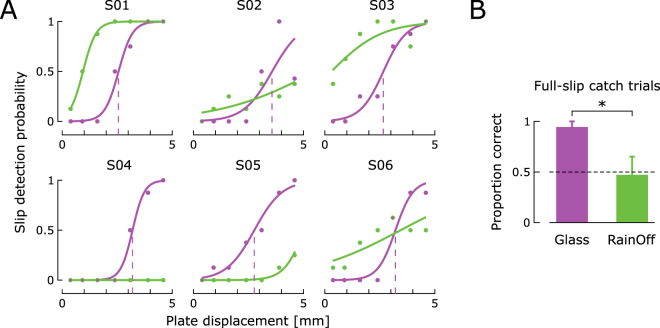
Experiment 2: Impaired slip detection in the low-friction condition. **(A)** Answers from all six subjects plotted against PD for the high- (purple) and low- (green) friction conditions. Subjects perceived fingertip slip consistently in the high-friction condition. However, in the low-friction condition, most subjects reported that they were not able to detect slippage and therefore answered randomly. Purple and green dots correspond to the average of all trials sharing the same PD within the corresponding condition. They are displayed for illustrative purposes only; psychometric curves were fitted on data from individual trials. **(B)** Results from full-slip catch trials. Bar graphs summarize the ability of subjects to detect fingertip slip when the plate was fully slipping under their fingertip. Data are displayed as the mean proportion of correct answers ± standard error across subjects. In the high-friction condition (glass), subjects’ performance was above 95%. In contrast, detection accuracy decreased to 47% (near the 50% chance level, dashed line) in the low-friction condition (RainOff), effectively reflecting the inability of subjects to detect slippage in this condition.

In the second experiment, two friction conditions were contrasted: a high-friction condition using bare glass as in Experiment 1, and a low-friction condition, obtained by covering the glass plate with RainOff. In both conditions, NF was kept constant at 5N for all trials. In the high-friction condition, we reproduced results from Experiment 1: subjects perceived fingertip slip during incipient slip (SR > 0). However, in the low-friction condition, most subjects reported that they did not perceive the plate slipping under their fingertip. Green traces in Fig. 3A show the answers from all subjects plotted against PD in the low-friction condition. In this condition, stick-to-slip transition was abrupt and SR reached zero for PD < 1 mm (see control experiment). Therefore, the plate was fully slipping under the subjects’ fingertip in most of the low-friction trials. In spite of this, subjects often failed to detect the plate slipping, e.g. S02 in Fig. 3A. This was confirmed by the subjects’ responses in full-slip catch trials (Fig. 3B) wherein the large PD (14 mm) always yielded a full slip between the fingertip and the plate. In full-slip catch trials performed on bare glass (high friction), subjects were able to detect slippage of the plate with more than 95% accuracy (Fig. 3B, purple). However, this ability was dramatically impaired and their detection accuracy dropped to 47% in the low-friction (RainOff) condition (Fig. 3B, green). Subject responses in full-slip catch trials differed significantly across the two conditions (paired t-test, t(5) = 2.79, p < 0.05). Catch trial responses are further described in the Supplemental Material.

### Control experiment

**Figure 4:**
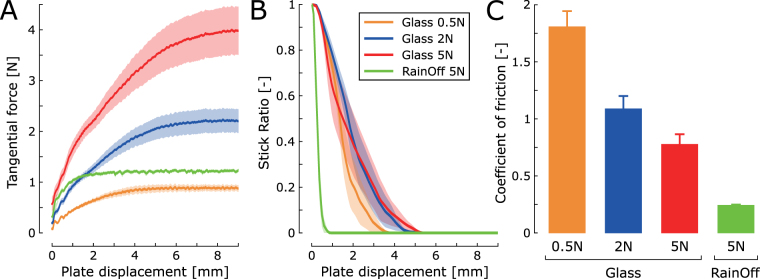
Control experiment testing finger pad mechanics. **(A)** TF and **(B)** SR plotted against PD for the following conditions: bare glass with a NF of 0.5 N (orange), 2 N (blue), and 5 N (red), and RainOff-covered glass with a NF of 5 N (green). Data are displayed as mean ± standard error across subjects. **(C)** Mean dynamic coefficient of friction ± standard error across subjects. Friction differed among all conditions (p < 0.001).

A control experiment was carried out to compare finger pad mechanics across experimental conditions to investigate why subjects could detect slip in the high-friction condition but not in the low-friction one. We included an additional very-low NF bare glass condition (NF = 0.5N) to investigate the mechanical behaviour of the finger pad in conditions similar to those employed by Srinivasan *et al*.^13^. For all trials, a large PD (9 mm) was used, sufficient to cause full slip and to allow TF to plateau. We measured the evolution of TF and SR as a function of PD (Fig. 4A,B) and measured the dynamic coefficient of friction between the fingertip and the plate in the four experimental conditions (Fig. 4C). A significant effect of experimental condition on the coefficient of friction was observed (one-way repeated-measures analysis of variance (ANOVA), F(3,15) = 109.27, p < 0.001), with significant differences in friction being observed among all four conditions (Bonferroni-corrected pairwise comparisons, p < 0.001).

In the low-friction (RainOff) condition, TF plateaued earlier than in the high-friction (bare glass) conditions (Fig. 4A), indicating that fingertip shearing was reduced in this condition. In addition, the lower friction made SR reach zero, i.e. the fingertip was fully slipping, for PD < 1 mm whereas the contact entered full slip for PD > 3 mm in high-friction conditions (Fig. 4B). Together, these results indicate that fingertip volume deformation was reduced in the low-friction condition.

Finally, in the very-low NF condition (0.5N), TF and therefore fingertip shearing were lower than in the low-friction (RainOff) condition (Fig. 4A). However, the relationship between SR and PD was not dramatically affected by the reduction in NF and remained similar to that observed in other bare-glass conditions (Fig. 4B).

## Discussion

In this study, we investigated how humans perceive slippage of their fingertip under tangential loading. In two experiments, the index fingertip of subjects was stroked passively by a smooth glass plate, while the evolution of slip at the contact between the fingertip and the plate was imaged through the transparent glass. We measured the subjective threshold of slip detection as a function of several predictors: stick ratio (SR), plate displacement (PD), and tangential force (TF) applied to the plate by the fingertip. We found that subjects perceived slippage of a bare glass plate stroking their fingertip well before full slip, and that they could do so by relying either on the extent of partial slip under their fingertip, as measured with SR, or on PD, or on a combination of both. In addition, TF was found not to be a reliable predictor of the perception of slippage. Moreover, the subjects were unable to detect the plate slipping under low contact friction, even though NF was the same as with the bare glass plate. Finally, under low contact friction we also observed a reduction of the amount of fingertip shearing.

We used the psychophysical method of constant stimuli to measure the slip detection thresholds of the tested subjects. The tactile stimuli such as the ones used here cannot be presented in parallel. Therefore, subjects were exposed to a single stimulus and then had to report whether or not they felt the plate slip under their fingertip. This 1-interval scheme has the advantage of being independent from the subject’s reaction time. In addition, subjects did not have to memorize stimuli to compare them with future stimuli. However, 1-interval methods are bias-prone when used alone. Therefore, we introduced additional procedures to control for response bias. First, we controlled for the bias that would be related to stimulus direction by making the plate move in the ulnar or radial direction in a random fashion, with an equal total number of trials in each direction. We tested for an effect of stimulation direction on subject responses and found none (see Results). In addition, data have been analysed using paired t-tests across conditions, making our results robust against constant response bias in individual subjects. Finally, we used catch trials to check that subjects were able to detect correctly the extreme values of the stimuli, i.e. no slip or full slip. Subjects performed well on these catch trials, as detailed in the Supplemental Material. Therefore, our control procedures made us confident in the quality of the collected data.

Previously, Srinivasan *et al*. reported that subjects did not detect slippage of a smooth glass plate pressed with a NF of 0.2N^13^, a force level that was an order of magnitude below those used in our study, therefore causing far less fingertip deformation, as demonstrated in our control experiment. Together, these results show that fingertip slip detection is impaired by either a low NF or a low contact friction. In addition, when subjects could not detect slippage of the smooth plate, volume deformation of the fingertip, i.e. the amount of fingertip shearing, was lower than when they were able to detect it. Therefore, it seems that sufficient fingertip deformation is needed to detect slip on smooth surfaces.

In light of these observations, we propose that, in addition to the vibration-based mechanism allowing subjects to detect slippage of rough surfaces discussed by Srinivasan *et al*.^13^, there also exists a deformation-based mechanism enabling slip detection on smooth surfaces. Fingertip deformation is two-fold. On the one hand, the stroking plate causes fingertip shearing, i.e. volume deformations. As long as the finger is not slipping fully against the plate, these deformations are related to PD. Indeed, before full slip some parts of the finger pulp accompany the movement of the plate. Therefore, an increase in PD leads to an increase in fingertip shearing, i.e. an increase in its volume deformation. On the other hand, fingertip skin in contact with the plate undergoes systematic surface deformations during fingertip shearing due to partial slips, as measured by SR^11^. Therefore, we believe that PD and SR, which are respectively proxies of volume and surface deformations during incipient slip (i.e. before full slip), both play a role in the hypothesized slip detection mechanism based on fingertip deformations. In addition, we hypothesize that the vibration- and deformation-based neural mechanisms are not mutually exclusive, but instead provide two complementary modes of detecting fingertip slips.

In addition, the fact that subjects perceived slip before full slip has functional implications for object manipulation. Indeed, this early slip detection may allow us to anticipate full slip and therefore to correct grip force before object dropping. It is therefore reasonable to hypothesize that a motor control mechanism could use partial slips to adjust grip force during object manipulation. Indeed, human manipulation abilities critically rely on tactile signals sent to the central nervous system by tactile afferents innervating the fingertips^15^. The lack of such feedback has a dramatic impact on manipulation performance and impedes our ability to adjust grip force in response to unexpected perturbations^16,17^. Johansson and Westling reported automatic grip force adjustments in response to sudden accelerations attributed to brief slips between fingertips and an object^18^. In addition, they observed similar adjustments in the absence of an overall slip of the object. They hypothesized that these adjustments were triggered by partial slips, though they did not measure them directly^14^.

We intend to examine the partial-slip-based control mechanism with an instrumented manipulandum combining sensing of the forces applied by the fingers to the object and imaging of the fingertip contact, which would enable us to observe partial slips during dexterous manipulation and to determine how they may be involved in the adjustment of grip force. Interestingly, Ho and Hirai recently demonstrated the applicability of a partial-slip-based mechanism to control robotic grippers. They developed a robotic controller adjusting grip force based on the level of partial slip, and demonstrated with simulations that it was able to prevent full slip at the fingertip-object contact^19^. Their controller provides a robust and efficient grasping control by maintaining grip force just above the minimum level needed to avoid object dropping, similar to what is observed in human manipulation^20^.

To summarize, this study provides evidence that partial slips play a significant role in fingertip slip perception. In addition, it is reasonable and functionally relevant to hypothesize that the central nervous system could rely on partial slips to adjust grip force during object manipulation.

## Methods

### Participants

Ten healthy human subjects (age range 23–35 years; three women) participated in the first experiment. Six of them also participated in the second experiment and in the control experiment. All subjects provided written informed consent to undergo the procedure, which was approved by the ethics committee at the host institution (Institute of Neuroscience, Université catholique de Louvain, Brussels, Belgium). The investigation conformed to the principles of the Declaration of Helsinki and experiments were performed in accordance with relevant guidelines and regulations. Participants were blindfolded during the procedure and there were no auditory cues that would allow them to perceive robot movements.

### Apparatus and data collection

We used a robotic platform based on an industrial robot able to move plates in three orthogonal directions (Denso Robotics, Japan) to place a transparent glass plate in contact with the right index fingertip of subjects and to move it tangentially in the radial or ulnar direction (Fig. 1B). The surface of the glass plate was optically flat and smooth. The plate was cleaned manually with a microfiber cloth before every trial. For each trial, the glass plate was first loaded perpendicularly against the finger pad at a given NF, which was kept constant throughout the trial (Fig. 1A). Once NF stabilized, the plate moved tangentially over a given distance with a constant speed of 5 mm/s. After a short pause, the plate was retracted from the finger pad. We used two force transducers mounted on each side of the glass plate to measure the forces applied to it by the subject’s fingertip (ATI force sensors, ATI Industrial Automation, Apex, NC). The plate position and forces exerted on it were recorded at 1 kHz. In addition, we imaged the fingerprints in contact with the transparent glass plate with a high-speed (50fps), high-resolution (1200dpi) camera and a coaxial light source. This custom fingerprint imaging apparatus allowed us to compute the proportion of the contact slipping against the glass (Fig. 1C). The robotic platform and optical apparatus are described in greater detail elsewhere^10,11^.

### Experimental procedures

We conducted two psychophysical experiments with constant stimuli during which a glass plate was moved tangentially in the radial or ulnar direction under the right index fingertip of subjects in successive trials. At the end of each trial, i.e. after the plate was retracted from the finger pad, subjects were asked to report whether they detected the plate slipping under their fingertip. The fact that subjects gave their answer after the end of the trial ensured that the results were not biased by reaction time. We tested seven PD levels, ranging linearly from 0.5 mm to 5 mm. The duration of the pause at the end of each trial was adjusted to ensure a constant period of 1.4 s between the onset of the horizontal plate movement and plate unloading (Fig. 1E).

The first experiment consisted of two NF conditions (2N vs. 5N). Each stimulus was repeated eight times for a total of 112 trials per subject (2 NF levels x 7 PD levels x 8 repetitions). Trial presentation order was randomized. In addition to regular trials, catch trials were performed every eighth regular trial, for a total of 16 catch trials. These catch trials consisted of either no PD (4 trials), or 14-mm PD (12 trials), sufficiently large to ensure full slip between the fingertip and the plate. The total number of trials was thus 128 (112 regular trials + 16 catch trials). The duration of the full experiment was about 1.5 hour.

In the second experiment, two friction levels (high vs. low) were tested and NF was kept constant at 5N. In the high-friction condition, the same bare glass plate was used as in the first experiment. In the low-friction condition, the glass plate was covered with RainOff, a transparent hydrophobic coating that reduces the surface-skin friction strongly (Arexons, Italia). Without RainOff, the dynamic coefficient of friction between the fingertip skin and the glass plate was close to 1, whereas with RainOff it decreased to 0.3. All trials from the high-friction (bare glass) condition preceded all trials from the low-friction (RainOff) condition because RainOff residue is difficult to remove from skin between trials. The number of trials was the same as in the first experiment (8 repetitions of 7 PD levels for 2 conditions = 112 regular trials, accompanied by 16 catch trials as described above, for a total of 128 trials).

### Control experiment

The goal of this experiment was to characterize finger pad mechanics under the conditions tested in the two main experiments in order to investigate why subjects failed to detect fingertip slip in the low-friction condition of Experiment 2. The control experiment consisted of four mechanical conditions: bare glass with a NF of 0.5N, 2N, or 5N, and glass covered with RainOff with a NF of 5N. All other parameters were the same as in the main experiments, except for PD amplitude, which was increased to 9 mm to allow TF to plateau in all conditions. Each condition was tested 5 times, totalizing 20 trials per subject. The results of this experiment depended only on finger pad biomechanics and were highly reproducible.

### Data processing and statistical analyses

Position and force signals were low-pass filtered using a fourth-order, zero-phase-lag Butterworth filter with an 80Hz cut-off frequency. In the control experiment, we computed the dynamic coefficient of friction as the TF:NF ratio during the TF plateau.

We tracked the position of fingerprint features in sequences of fingertip contact images and computed their displacement with respect to the glass surface. By spotting the features moving relative to the glass, we could measure the proportion of the fingertip-plate contact undergoing slippage and compute SR as the ratio of the no-slip area to the whole contact area between the fingertip and the plate. When slip occurs, SR falls progressively from 1 (no slip) to 0 (full slip)^8–10^.

Subjects’ answers were analysed using binary logistic regression. Individual subject’s responses were modelled using the psychometric function given in Eq. 1 (generalized linear model):

1 $${{\rm{\Phi }}}^{-1}[P({y}_{i}=1)]={\beta }_{0}+{\beta }_{1}{x}_{i}$$

where ${{\rm{\Phi }}}^{-1}$ is the probit link function, i.e. the inverse function of the cumulative Gaussian distribution, ${y}_{i}$ is the binary response of the subject for the trial $i$ (1 if slip perceived and 0 if not), $P({y}_{i}\,=\,1)$ is the slip detection probability, and ${x}_{i}$ is the stimulus value at the end of the trial $i$, i.e. at the end of the horizontal movement of the platform. Three different predictors were considered: plate displacement (PD), stick ratio (SR) and tangential force (TF). Psychometric functions were fitted separately to the responses of each participant in each experimental condition and against the three predictors. From each model, we extracted the *slip detection threshold* as described in Eq. 2:

2 $$slip\,detection\,threshold=-\frac{{\beta }_{0}}{{\beta }_{1}}$$

This threshold represents the corresponding predictor value leading to a slip detection probability of 50%. This means that for predictor values larger than the threshold, subjects detected slip with a probability larger than chance.

Paired t-tests were carried out to detect differences in slip detection thresholds between pairs of experimental conditions. A one-way ANOVA with repeated measures was used to detect differences in friction between experimental conditions in the control experiment. Bonferroni-corrected paired t-tests were used to perform post-hoc multiple comparisons. The Kolmogorov-Smirnov test was used to test for an effect of stimulus direction on subject responses. P-values < 0.05 were considered significant. All data were processed and analysed in MATLAB (The MathWorks Inc., Natick, MA) and R (R Project for Statistical Computing, https://www.R-project.org/). The datasets generated during and/or analysed during the current study are available from the corresponding author on reasonable request.

## Electronic supplementary material

Supplemental Information
