# Supplementary material for: Perception of partial slips under tangential loading of the fingertip
Source: Sci Rep. 2018 May 4;8:7032. doi: 10.1038/s41598-018-25226-w (PMC5935679; doi:10.1038/s41598-018-25226-w)
Supplement: Supplementary file 1 — Supplemental Information [file 41598_2018_25226_MOESM1_ESM.docx]

# Perception of partial slips under tangential loading of the fingertip

## Supplemental Information

Allan Barrea^1,2^, Benoit P. Delhaye^4^, Philippe Lefèvre^1,2^, Jean-Louis Thonnard^1,3,^*

^1^ Institute of Neuroscience, Université catholique de Louvain, Brussels (B-1200), Belgium.

^2^ ICTEAM Institute, Université catholique de Louvain, Louvain-la-Neuve (B-1348), Belgium.

^3^ Physical and Rehabilitation Medicine Department, Cliniques Universitaires Saint-Luc, Brussels (B-1200), Belgium.

^4^ Department of Organismal Biology and Anatomy, University of Chicago, Chicago, Illinois.

* Correspondence should be addressed to J.-L.T. ([jean-louis.thonnard@uclouvain.be](mailto:jean-louis.thonnard@uclouvain.be)).

# Supplemental Information

## Catch trial responses

The catch trials consisted of either no plate displacement or of a large plate displacement (14mm) guaranteeing full slip between the fingertip and the plate. In the zero-displacement catch trials, subjects always correctly reported the absence of slippage between their fingertip and the plate, presumably due to the absence of tangential force. Suppl. Fig. 1 presents responses of subjects to the full-slip catch trials. In these trials, subjects detected slipping in the two conditions of Experiment 1 and in the high-friction condition of Experiment 2, which were all performed on bare glass. However, in the low-friction (RainOff) condition of Experiment 2, performance was reduced dramatically near chance level (50%).


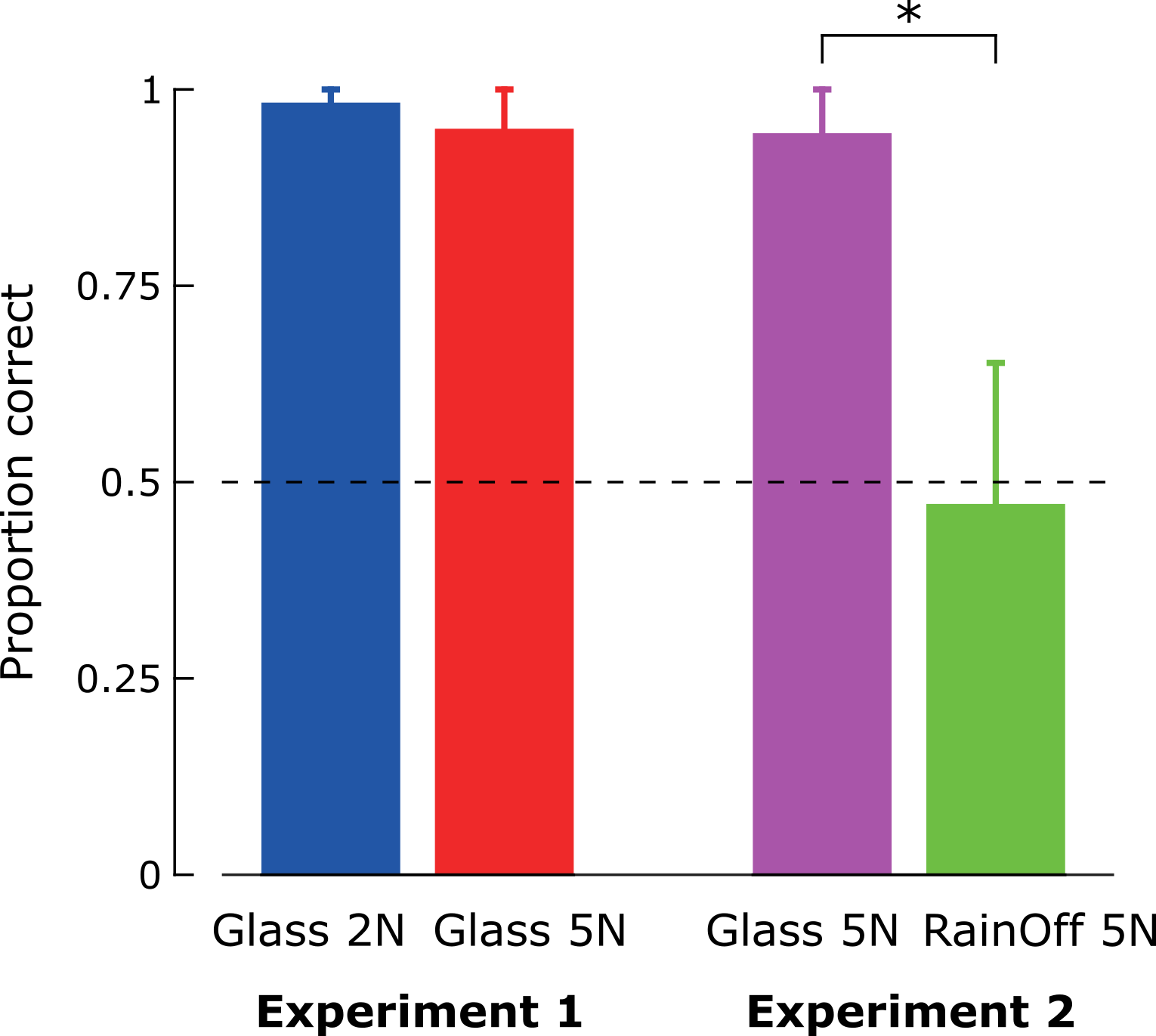


**Supplemental Figure 1 – Full-slip catch trial responses.** Proportions of correct answers are displayed as mean ± standard error for each condition. Subjects detected full slip between their fingertip and the plate in all conditions performed with the bare glass plate, but their performance was poor in the low-friction condition of Experiment 2, in which the plate was covered with RainOff. The asterisk denotes a significant reduction in the proportion of correct answers (p < 0.05). The horizontal dashed line indicates chance level (50%).
